# Retraction: Short polyethylene glycol chains densely bound to soft nanotube channels for inhibition of protein aggregation

**DOI:** 10.1039/d4ra90125h

**Published:** 2024-10-17

**Authors:** N. Kameta, T. Matsuzawa, K. Yaoi, M. Masuda

**Affiliations:** a Research Institute for Sustainable Chemistry, Department of Materials and Chemistry, National Institute of Advanced Industrial Science and Technology (AIST) Tsukuba Central 5, 1-1-1 Higashi, Tsukuba Ibaraki 305-8565 Japan n-kameta@aist.go.jp +81-29-861-4545 +81-29-861-4478; b Bioproduction Research Institute, Department of Life Science and Biotechnology, AIST Tsukuba Central 6, 1-1-1 Higashi, Tsukuba Ibaraki 305-8566 Japan

## Abstract

Retraction of ‘Short polyethylene glycol chains densely bound to soft nanotube channels for inhibition of protein aggregation’ by N. Kameta *et al.*, *RSC Adv.*, 2016, **6**, 36744–36750, https://doi.org/10.1039/C6RA06793J.

We the named authors hereby wholly retract this *RSC Advances* article due to the fact that the paper has wrong electron microscopy images in Fig. 2 and S3 on the part of the first author, who is affiliated with the National Institute of Advanced Industrial Science and Technology (AIST).

Fig. 2a should have displayed the TEM image of the PEG8-NT (the nanotubes composed of lipid 1, lipid 2 and glyPEG8). However, the first author posted the TEM image of the nanotubes composed of lipid 1.

Fig. S3a–c should have displayed TEM images of the PEG2-NT (the nanotubes composed of lipid 1, lipid 2 and glyPEG2), the PEG4-NT (the nanotubes composed of lipid 1, lipid 2 and glyPEG4) and the PEG12-NT (the nanotubes composed of lipid 1, lipid 2 and glyPEG12). However, the first author posted TEM images of PEG8-NT in those figures.

The authors respectfully retract this paper, because these events were determined to amount to scientific misconduct and the retraction of this paper was recommended by AIST. AIST verified that the first author was responsible for the misconducts and no other co-authors were engaged in them.

Signed: Tomohiko Matsuzawa, Mitsutoshi Masuda, Katsuro Yaoi, Naohiro Kameta

Date: 3rd October 2024

Retraction Endorsed by Laura Fisher, Executive Editor, *RSC Advances*

